# Parallel morphological evolution and habitat‐dependent sexual dimorphism in cave‐ vs. surface populations of the *Asellus aquaticus* (Crustacea: Isopoda: Asellidae) species complex

**DOI:** 10.1002/ece3.8233

**Published:** 2021-10-20

**Authors:** Gergely Balázs, Anna Biró, Žiga Fišer, Cene Fišer, Gábor Herczeg

**Affiliations:** ^1^ Behavioural Ecology Group Department of Systematic Zoology and Ecology ELTE Eötvös Loránd University Budapest Hungary; ^2^ Department of Biology Biotechnical Faculty University of Ljubljana Ljubljana Slovenia

**Keywords:** adaptation, colonization, parallel evolution, sexual dimorphism, subterranean, troglomorphy

## Abstract

Studying parallel evolution (repeated, independent evolution of similar phenotypes in similar environments) is a powerful tool to understand environment‐dependent selective forces. Surface‐dwelling species that repeatedly and independently colonized caves provide unique models for such studies. The primarily surface‐dwelling *Asellus aquaticus* species complex is a good candidate to carry out such research, because it colonized several caves in Europe. By comparing 17 functional morphological traits between six cave and nine surface populations of the *A. aquaticus* species complex, we investigated population divergence in morphology and sexual dimorphism. We found habitat‐dependent population divergence in 10 out of 17 traits, likely reflecting habitat‐driven changes in selection acting on sensory systems, feeding, grooming, and antipredator mechanisms. Sexual dimorphism was present in 15 traits, explained by sexual selection acting on male traits important in male–male agonistic behavior or mate guarding and fecundity selection acting on female traits affecting offspring number and nursing. In eight traits, the degree of sexual dimorphism was habitat dependent. We conclude that cave‐related morphological changes are highly trait‐ and function‐specific and that the strength of sexual/fecundity selection strongly differs between cave and surface habitats. The considerable population variation within habitat type warrants further studies to reveal cave‐specific adaptations besides the parallel patterns.

## Graphical abstract

Surface‐dwelling species that repeatedly and independently colonized caves such as Asellus aquaticus provide unique models to study parallel evolution. We measured and analyzed 17 functional morphological traits on 656 individuals from 15 cave and surface populations. We found that besides the expected habitat‐related morphological changes habitat type also affected sexual dimorphism.

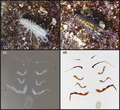

## 1 INTRODUCTION

A fundamental goal of evolutionary biology is to understand phenotypic variation in the wild. Spatial and temporal environmental variation results in variation in forces of natural selection, ultimately resulting in between‐population and between‐species adaptive phenotypic divergence (Endler, 1977, 1986). Comparison of organisms living in similar environments provides a particularly strong natural setup to link environment to phenotypic evolution, both in the case of similar adaptations appearing independently in different taxa (convergent evolution) or repeatedly and independently among different populations of the same species (parallel evolution) (Bolnick et al., 2018; Endler, 1986; Schluter et al., 2004). Caves with their (i) unique and constant environments, (ii) “island like” properties like simple communities and restricted gene flow, and (iii) the similar environmental conditions in geographically separate locations creating independent replicates offer a naturally replicated experiment for understanding the process of evolution (Culver et al., 1995; Culver & Pipan, 2009; Mammola, 2019; Romero, 2009, 2011). Repetitive morphological adaptations were recognized early in the history of cave biology and referred as troglomorphism by Christiansen (1962). Later, troglomorphism was extended to all cave‐related phenotypic changes (Christiansen, 1965). These changes can be classified into two groups: regressive and progressive adaptations (for review, see Christiansen, 2012). A common regressive adaptation is the reduction of the visual system, while the increased development of extra‐optic sensory systems is a prime example of a progressive troglomorphy (Pipan & Culver, 2012).

Some morphological modifications of subterranean animals are conspicuous, such as the lack of body pigmentation or the elongation of appendages (Pipan & Culver, 2012). However, even these frequent changes cannot be considered universal, as there are other limiting factors than the absence of light (which is universal in all caves, but see Badino, 2000; Mejía‐Ortíz et al., 2018), like limited food sources or the absence of daily and annual rhythmicity (Culver et al., 2010). Certain traits evolve under multiple, sometimes even opposing selection pressures stemming from the subterranean environment. A prime example of this is the relative appendage size of cave arthropods. Generally, the absence of light selects for longer appendages as they can aid extra‐optic sensing to compensate the reduction of visual sensory structures (Culver & Pipan, 2009, hereafter “elongation hypothesis”). On the contrary, the utilization of small spaces like fissures, or aquatic habitats with high water velocity selects against long appendages as they would be unfavorable for locomotion or even lethal in case of drifts during floods (Kralj‐Fišer et al., 2020). Although it is believed that the key driver of these changes is the subterranean environment, other factors such as sexual selection might affect the development and magnitude of troglomorphic traits. Sexual selection is broadly studied in surface‐dwelling animals and is known to cause behavioral, physiological, and morphological differences between males and females (Andersson, 1994; Clutton‐Brock, 2007; Fairbairn et al., 2007; Shine, 1989). However, most studies of parallel evolution, or generally adaptive divergence, do not consider sexual dimorphism, but rather pool the sexes or base the analyses on one sex only, even though sexual dimorphism can represent phenotypic divergence comparable to adaptive divergence between habitats (Butler et al., 2007; Hendry et al., 2006; Oke et al., 2019).

To understand the general rules of evolutionary changes following the colonization of the subterranean environment, we need to study appropriate models. Such models are provided by surface species, which tend to repeatedly and independently colonize subterranean habitats. Prime examples are the fish *Astyanax mexicanus* de filipi, 1853, which was a subject to cave adaptation several times independently as its different populations got isolated in caves (Wilkens, 1988) and the aquatic amphipod *Gammarus minus* say 1818 with several surface‐ and cave‐dwelling populations, providing considerable variation among and within populations for morphological and phylogenetic comparisons (Carlini et al., 2009; Culver et al., 1995). Lately, another model taxon emerged, the primarily surface‐dwelling freshwater isopod *Asellus aquaticus* (linnaeus, 1758) species complex (Protas & Jeffery, 2012). It is widespread in the Western Palearctic region (Verovnik et al., 2004, 2005) and has successfully colonized caves in Europe on independent occasions (Pérez‐Moreno et al., 2017; Verovnik & Konec, 2019). Colonization of European caves by *A. aquaticus* and the isolation of the subterranean populations happened relatively recently (Pérez‐Moreno et al., 2017; Verovnik et al., 2003). Distinct cave populations have undergone parallel evolution resulting in similar cave‐related phenotypes and differ considerably from surface populations (Konec et al., 2015; Verovnik et al., 2004). Cave *A. aquaticus* populations show typical adaptations such as loss of pigmentation and reduction of eyes (Protas et al., 2011; Verovnik & Konec, 2019). Further, other troglomorphic adaptations (e.g., elongation of various appendage articles) have been found in three cave populations based on studies only including males (see Prevorčnik et al., 2004; Turk et al., 1996). Surface populations of the species complex are known to be sexually dimorphic in various traits including body size (Adams et al., 1985), length of antenna II (Blasdent, 1965), size and shape of pereopod I and pereopod IV (Bertin et al., 2002), and the length of pereopod VII (Blasdent, 1965). Contrary to this, there is only sporadic information on sexually dimorphic traits in case of the cave populations, mostly appearing in taxonomical descriptions (Turk‐Prevorčnik & Blejec, 1998; Verovnik et al., 2009). According to a comprehensive review (Mammola et al., 2021), this is true for all subterranean model species. Taken together, there is a need for a large‐scale study on a model species with the inclusion of both sexes from a large set of subterranean populations examining functionally relevant morphological traits to reveal parallel morphological cave adaptations and sexual dimorphism.

In the present study, we aimed to find signs of parallel morphological evolution between cave and surface *A. aquaticus* populations. We studied six cave populations representing independent cave colonizations according to previous genetic studies (Konec et al., 2015; Pérez‐Moreno et al., 2017; Verovnik et al., 2004, 2009) and contrasted them to nine surface populations chosen from the cave populations’ geographical proximity. We analyzed 17 functional morphological traits. The chosen traits cover a wide range of functions including locomotion, mating, and sensing. We included individuals of both sexes to see whether environmental selection conflicts sexual selection during cave adaptation. We addressed two questions. First, we asked what the main patterns in the cave––surface morphological divergence are. Second, we asked what the main patterns in the cave–surface divergence in morphological sexual dimorphism are. Owing to the large number of studied traits with various functions, we did not set up detailed hypotheses/predictions for all traits. However, we expected (i) elongation of structures bearing extra‐optic (chemosensory, mechanosensory) sensors and (ii) sexual dimorphism in traits with sex‐dependent roles. Finally, we specifically explored whether the level of sexual dimorphism is habitat dependent.

## 2 MATERIALS AND METHODS

### 2.1 Study system

The taxonomic status of *Asellus* populations in Europe is only partially resolved. Currently, there are two species (*A. aquaticus* Linnaeaus, 1758 and *A. kosswigi* Verovnik et al., 2009) and numerous subspecies formally described (Karaman, 1952; Racovitza, 1925; Schneider, 1887; Sket, 1965; Turk‐Prevorčnik & Blejec, 1998). Moreover, unilocus delimitation methods imply that there may be more species within the *A. aquaticus sensu lato* species complex (Sworobowicz et al., 2015, 2020). However, all these taxa are nested within the nominal *A. aquaticus*, indicating multiple divergences or ongoing speciation from the nominal *A. aquaticus* that proceeded to a different extent. For simplicity, we will refer to all sampled populations as “*A. aquaticus*” in the paper.

**FIGURE 1:**
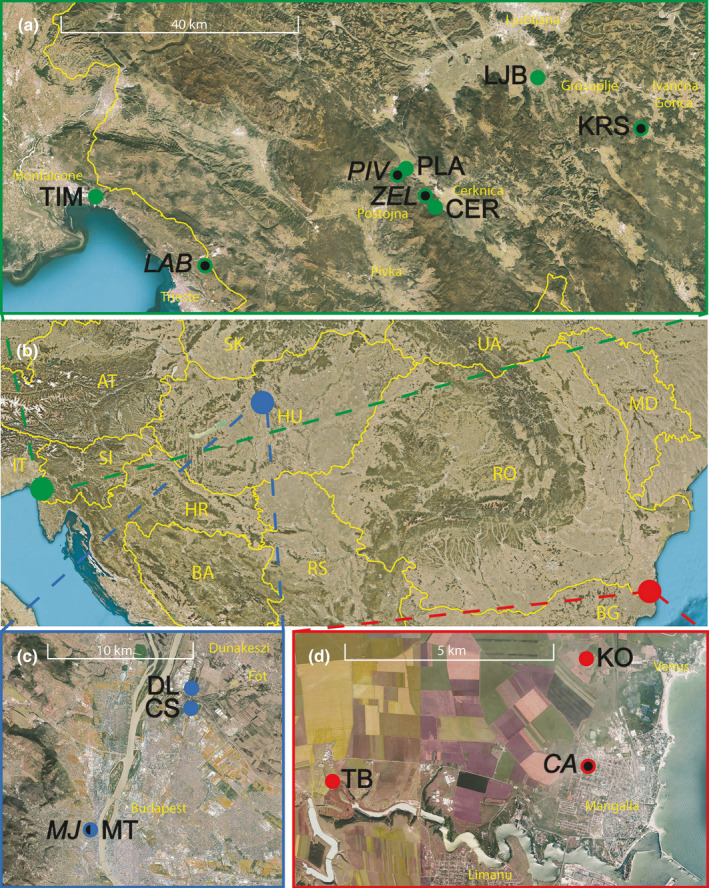
Geographical distribution of sampling locations. (a) Slovenia and Italy, (b) Central Europe, (c) Hungary, (d) Romania. Filled circles denote surface populations; filled circles with black dots denote cave populations. For population abbreviations, see Table 1

In this study, six cave–surface population pairs were collected from four countries (Table 1; Figure 1). As Hungarian and Romanian cave populations live in thermal and sulfidic caves, respectively, the surface counterparts comprised surface populations from thermal and sulfidic surface waters. To account for the effects of temperature and sulfide, we included three extra surface non‐thermal and non‐sulfidic populations. In the following text, we will use population abbreviations explained in Table 1. In five population pairs, there is no physical barrier between the cave and surface habitats as they inhibit the same sinking river (*LAB*‐TIM, *PIV*‐PLA, *ZEL*‐CER; abbreviations for cave populations are italicized) or the same groundwater–surface water continuum (*MJ*‐MT, *CA*‐KO). In case of the *KRS*‐LJB population pair, we collected surface individuals from the closest geographical location, as there is no population inhibiting the surface part of the sinking river. The extra surface populations from Romania (TB) and Hungary (CS, DL) were collected from hydrologically distinct but geographically close habitats. For more details about the sampled populations, see Electronic Appendix 1.

**TABLE 1:** Studied populations of the *Asellus aquaticus* species complex. Sample size is specified as the number of individuals used in the final analyses

ID	Country	Locality	Habitat	GPS coordinates	Female (no.)	Male (no.)	Collection date
*MJ*	Hungary	Molnár János Cave	Cave	47.518°N, 19.03608°E	17	26	2018. Aug. 17.
MT	Hungary	Malom Lake	Surface	47.518277°N, 19.035999°E	18	22	2018. Aug. 17.
CS	Hungary	Csömör Stream	Surface	47°35′35.03″N 19°07′21.78″E	14	19	2018. Aug. 17.
DL	Hungary	Dunakeszi Lake	Surface	47°36′23.15″N 19°07′24.63″E	16	20	2018. Aug. 17.
*CA*	Romania	Dimitru Ana Well	Cave	43°49′23.59″N, 28°34′01.45″E	17	27	2018. Jun. 06.
KO	Romania	Kara‐Oban Lake	Surface	43°50′46.0″N 28°33′59.1″E	9	11	2018. Jun. 06.
TB	Romania	Spring at Baile Turcesti	Surface	43°49′12.15″N 28°29′28.26″E	11	13	2018. Jun. 06.
PLA	Slovenia	Planina Polje	Surface	45°49′56.2″N 14°15′30.0″E	28	28	2018. Oct. 18.
*PIV*	Slovenia	Pivka Channel of Planina Cave	Cave	45°49′11.6″N 14°14′44.4″E	27	24	2018. Oct. 18.
CER	Slovenia	Cerknica Polje	Surface	45°46′23.0″N 14°19′31.2″E	29	29	2018. Oct. 18.
*ZEL*	Slovenia	Zelške Cave	Cave	45°47′26.4″N 14°18′12.6″E	24	28	2018. Oct. 18.
LJB	Slovenia	Ljubljana Moors	Surface	45°58′02.9″N 14°32′52.0″E	28	28	2018. Oct. 18.
*KRS*	Slovenia	Krška Cave	Cave	45°53′24.0″N 14°46′16.5″E	24	26	2018. Oct. 18.
TIM	Italy	Timavo Spring	Surface	45°47′15.8″N 13°35′28.7″E	26	28	2018. Oct. 18.
*LAB*	Italy	Labodnica Cave (Grotta di Trebiciano)	Cave	45°41′04.1″N 13°49′42.9″E	10	29	2018. Oct. 18.
Total					**298**	**358**	

### 2.2 Studied traits

**FIGURE 2:**
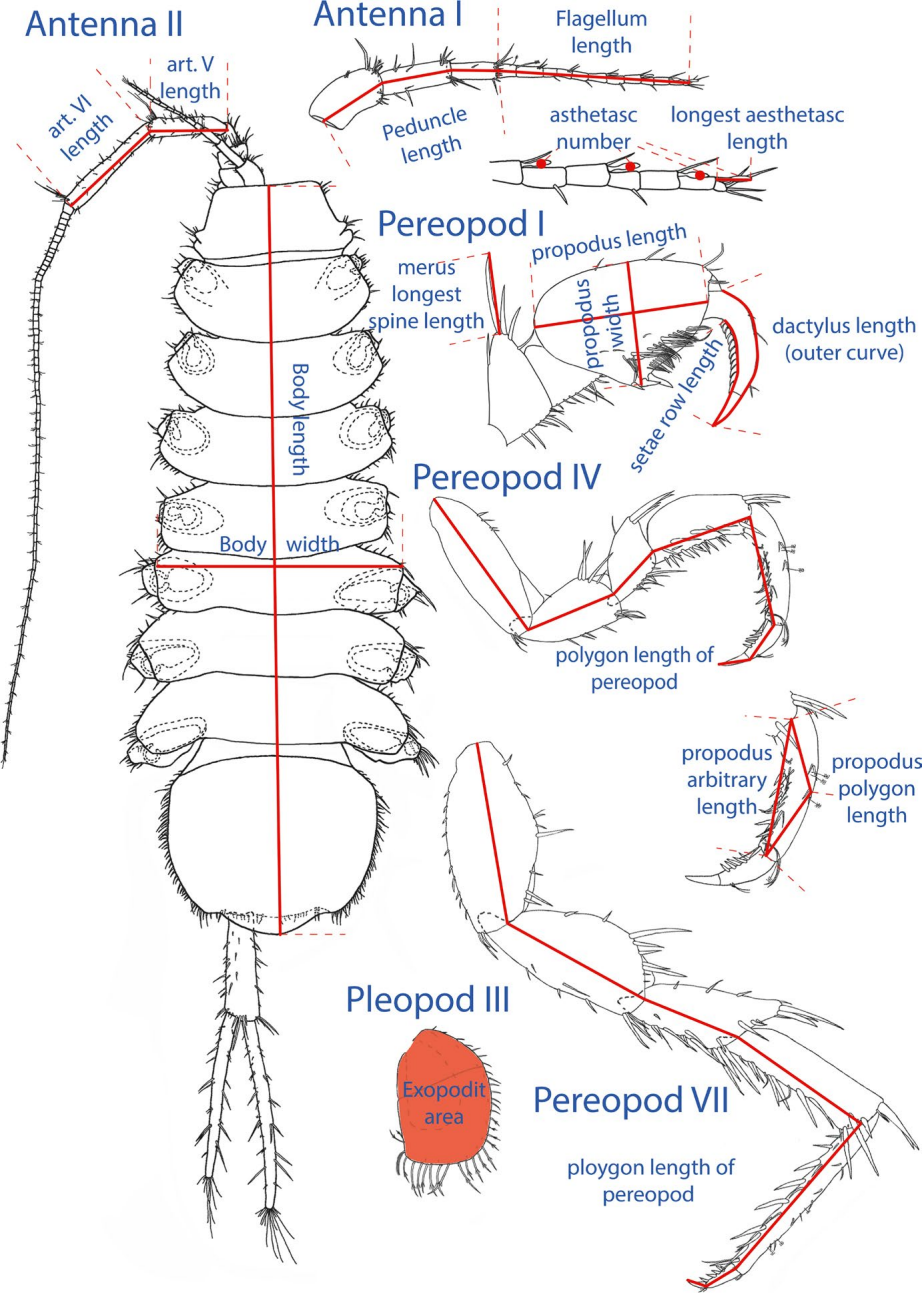
Drawing of *Asellus* body and appendages used in the study. Measurements and their landmarks are indicated in red. Original drawing by Simona Prevorčnik

The measured traits and landmarks used for the measurements are depicted in Figure 2. We used body length (as a body size proxy) only to express all other measured traits in relative terms and focused on their body size‐corrected variation. Body size of *A. aquaticus* is sexually dimorphic (Hay, 1999); a detailed analysis on body size and sexual size dimorphism variation on the specimens used in this study revealed no habitat‐dependent patterns (results to be published separately). Relative body width correlates positively with fecundity (Ridley & Thompson, 1979; Vick & Blum, 2010) and can also be used as a proxy for body elongation (Konec et al., 2015).

Antenna I was measured due to its extra‐optic (chemosensory) function (Heimann, 1984). The peduncle and the flagellum of antenna I were measured separately, as elongation of the articles might occur separately. We also counted the number and measured the maximal length of the aesthetascs (chemosensory sensillae) on the flagellum. Antenna II was measured due to its extra‐optic (mechanosensory) function (Culver et al., 1995). Longer antenna in some invertebrate species enhance mate detection (Hanks et al., 1996; Lefebvre et al., 2000). This might be also true for *A. aquaticus*, as Bertin and Cézilly (2003) reported that in its surface populations, males with longer antenna II have increased pairing success. As a proxy for antenna II size, we measured the length of the 5^th^ and the 6^th^ article of the peduncle separately. We did not measure the length of the flagellum because it tends to break easily and detecting the break is challenging, which would make total antenna II length incomparable across individuals (Prevorčnik et al., 2004). Even though studies about antenna II found that the preferential breakage point is just below the 5^th^ peduncle article (Maruzzo et al., 2007), we found that in most cases at least one of the sides is intact till this point.

The pereopod I distalmost articles, that is, the dactylus and the propodus, jointly form an apparatus for seizing and handling. This apparatus is used in feeding, aggressive displays, mating, and grooming. However, the two articles seem to have different importance in different functions. Propodus (subdistal article) that we measured as length and width, seems to be important in feeding, aggressive behavior, and mate guarding (Bertin et al., 2002), whereas dactylus (distalmost article) with its special carpal brush of setae situated on its inner curve seems to be important in antenna II grooming (Bauer, 2013). We measured the dactylus seta row length, and the length of the outer curve as a proxy for dactylus length. The length of the longest spine‐like setae on the merus of pereopod I (hereafter spine) was also measured. Even though the spine's function is not yet proven, an antipredator function has been shown for similar traits in other crustaceans such as atyid shrimps (Jugovic et al., 2010) and cladocerans (Boeing et al., 2006; Weiss et al., 2012).

Pereopod IV is used for locomotion in females, while it is modified in males for grasping and holding the female during precopulatory mate guarding (Adams et al., 1985; Ridley & Thompson, 1979). Like other pereopods, it also has a mechanosensory function in both sexes. Visually, females tend to have longer propodus than males, while males have a more curved propodus. We measured the total length of pereopod IV, and the arbitrary (the distance between the proximal and distal article point) and polygon length (another point on the dorsal article margin was included halfway between the previous points) of the propodus of the pereopod IV.

Pereopod VII has a locomotor and mechanosensory function in both sexes. Longer pereopod VII might result in enhanced walking performance (Bertin et al., 2002; Kralj‐Fišer et al., 2020). The total length of pereopod VII was measured.

The exopodit of pleopod III is suggested to have a role in swimming (Alexander et al., 1995). Its respiratory and osmoregulatory function is not generally accepted (these are assigned to endopodites of all pereopods and to the exopodit area of pleopod IV and V) (Holliday, 1988; McLaughlin, 1980). However, being the largest exopodit, it might help in circulating the water around the respiratory and osmoregulatory surfaces of other appendages and around the marsupium in females, as suggested for other crustaceans (Dahl, 1977). Alternatively, it might serve directly as a respiratory surface (Prevorčnik et al., 2009). We measured the total surface of the exopodit of pleopod III (see below).

### 2.3 Sampling and slide preparation

A total of 776 individuals of surface and cave animals were collected primarily by using a hand water net and by washing off the rocky substrate, respectively. Cave diving was necessary in the Molnár János Cave, here we used a modified Sket‐bottle for sampling (Chevaldonné et al., 2008). In all cases, only adult animals (>3.5 mm; Bloor, 2010) were collected. After collection, we determined individuals’ sex by inspecting the gonopod morphology under a Zeiss Stemi 2000 stereomicroscope (Carl Zeiss AG, Oberkochen, Germany). Individuals were then stored in RNAlater (Thermo Fisher Scientific Inc., Waltham, Massachusetts, USA) in separate, marked vials at 4 °C. Whole‐body pictures were taken for the measurement of body length and width after which the individuals were placed back into the vials and stored at 4 °C until slide preparation.

**FIGURE 3:**
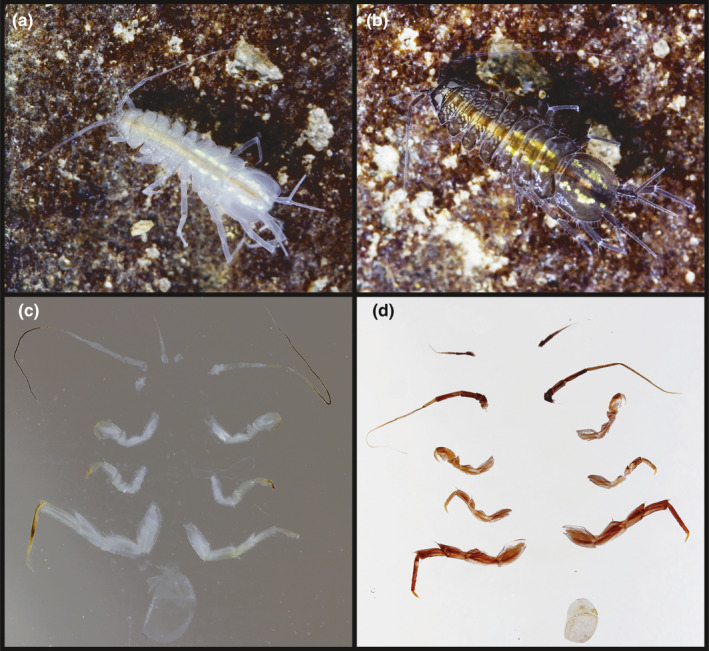
Cave (a) and surface (b) *Asellus aquaticus* in their natural habitat and slide‐mounted appendages of a cave (c) and surface (d) individual

For slide preparation, each individual was first put into distilled water for approximately 10 seconds. This was necessary as RNAlater occasionally crystalizes and can distort the images as a consequence. We carefully dissected antennae I, II and pereopods I, IV and VII from both body sides and the pleopod III exopodit from one body side. We mounted all appendages with dorsal side upward in Kaiser's glycerine gelatine (Merck KGaA, Darmstadt, Germany) on regular glass slides (VWR International, Radnor, Pennsylvania, USA) and covered them with coverslips (Figure 3).

### 2.4 Measurements

For body size measurements, a digital image was taken with a Canon 600D camera (Canon Inc. Tokyo, Japan) under standardized lighting and position, and with a millimeter scale bar as reference. We used the Tps Utility v.1.74 and Tps Dig2 v.2.30 software (https://life.bio.sunysb.edu/morph/index.html) to perform the measurements. Body length was measured from the apical line of the head to the end of the pleotelson while body width was measured at the widest part of the 5^th^ pereomere (Figure 2).

Slide‐mounted appendages were measured using a Zeiss Axioscope II microscope and the AnalySIS Program Package (Carl Zeiss AG, Oberkochen, Germany). For antenna I and II, pereopod I, IV, and VII, appendages from both body sides were measured. This was necessary, as like all arthropods, *A. aquaticus* tends to regrow its lost or broken appendages (Maruzzo et al., 2005). To minimize this unwanted source of variability, we kept only the values of the larger intact appendage for the analyses. The total area of the pleopod III exopodit was measured by circumscribing the surface area with 30 landmarks.

### 2.5 Statistical analyses

Altogether, we measured 17 morphological traits on 766 individuals. All animals with any missing data (~15% of the total dataset) were excluded from statistical analysis. Most of the missing data resulted from broken, regrown, or missing appendages. The final sample size was 656 individuals (298 females and 358 males). The measured variables are neither biologically nor statistically independent, and thus, they could not be tested directly one by one in independent tests. Two approaches were employed to overcome this problem. First, we performed a principal component analysis (PCA) to collapse the original variables into a smaller set of independent principal components (PCs) and used those in further univariate analyses. Second, we ran a multivariate linear model (mLM) to test for patterns in multivariate space, and upon significant effects, we ran univariate tests on the original variables. The two approaches are complementary. Applying the first, we tested how certain groups of traits are changing together, that is, revealed a latent pattern, but potentially lost information on changes in individual traits. Using the second approach, we investigated trait‐by‐trait patterns. All analyses were performed using SPSS 26.0 (IBM Corp., New York, USA).

A total of 16 metric and one meristic (aesthetascs number on antenna I) traits were analyzed. Metric traits are obviously size‐dependent. Additionally, the meristic trait also showed size dependence in a linear model built with aesthetascs number as a dependent variable, and population, body length, and their interaction as fixed effects (population: *F*_14, 627_ = 1.41; *p* = .14; body length: *F*_1, 627_ = 145.43; *p* < .001; population × body length: *F*_14, 627_ = 2.25; *p* = .006). For these reasons, we controlled for body size in all analyses. In the PCA, the residuals from linear regressions against body length were used, one linear regression for both sexes. In the mLM and subsequent univariate analyses, body length was included as a fixed effect (covariate) in the models.

To find the best structure in the PCA and to set the threshold values, we followed guidelines from Norman and Streiner (2008) and Tabachnick and Fidell (2013). We applied varimax rotation so that most original variables had strong loadings on one PC, and every PC had strong loadings from at least three original variables. We kept PCs only with eigenvalues > 1 and only considered factor loadings higher than 0.55. Further, we removed three variables (the length of the longest aesthetascs on antenna I and the lengths of the 5^th^ and 6^th^ article on antenna II) from the PCA, which did not load on any of the PCs or had strong loadings on more than one PC (thus these traits were analyzed only in the mLM approach). We analyzed the PC scores in linear mixed models (LMMs), with the given PC as dependent variable, habitat, sex, and their interaction as fixed effects and population nested in habitat as a random effect.

In the second approach, we built an mLM model with the 17 original variables as dependent variables, and habitat, sex, their interaction, and body length as fixed effects. Population nested in habitat was added as an extra fixed effect to capture additional variance structure of the data. Theoretically, it would be better to enter population nested in habitat as a random effect but fitting random effects in multivariate models are problematic at best. Since we were primarily interested in the significance of habitat and sex effects in the multivariate framework, allowing us to move toward the trait‐by‐trait univariate analyses, our model was sufficiently informative. Note that the above‐described mLM had qualitatively similar results when we removed the population effect, or when we built a model with population effect instead of habitat (data not shown). As the main effects (habitat, sex, and their interaction) were highly significant (see Results), we proceeded with univariate LMMs on the original variables. These LMMs were built with the given trait as dependent variable and habitat, sex, their interaction, and body length as fixed effects, and population nested in habitat as a random effect. Although aesthetasc number is a count variable, model residuals showed no deviation from normality after visual inspection of Q‐Q plots.

## 3 RESULTS

### 3.1 Approach 1: Principal component analysis followed by linear mixed models

**FIGURE 4:**
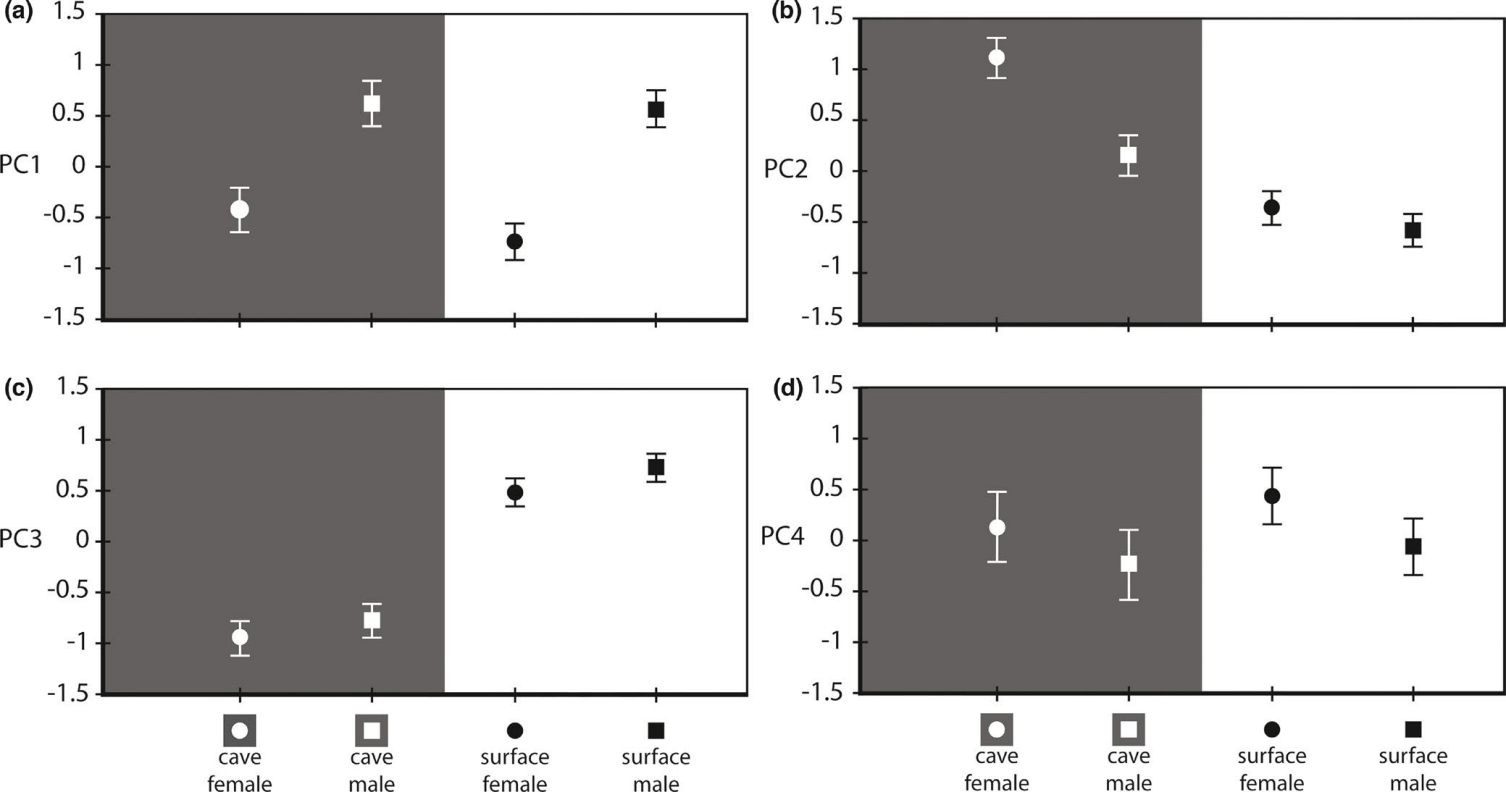
Results of linear mixed models ran on the four principal components (PCs). (a) PC1: significant sex and habitat ×sex effects. (b) PC2: significant habitat, sex, and habitat × sex effects. (c) PC3: significant habitat and sex effects. (d) PC4: significant sex effect. Least‐squares means ± standard errors are shown

The PCA resulted in four PCs, altogether describing 79.7% of the total variation (Table 2). The first PC (41.9% of variation) correlated positively with the length and width of the propodus of pereopod I, the setae row length, and the dactylus length of the same appendage and with the length of pereopod VII. The LMM on PC1 revealed significant sex effect and habitat ×sex interaction (habitat: *F*_1, 13_ = 0.40, *p* = .54; sex: *F*_1, 640_ = 577.56, *p* < .001; habitat ×sex: *F*_1, 640_ = 6.62, *p* = .01; Figure 4a). Males had higher scores than females and sexual dimorphism was more pronounced in surface populations.

**TABLE 2:** Component matrix (factor loadings) of the principal component analysis after varimax rotation

Trait	PC1	PC2	PC3	PC4
Peduncle (ant I)	0.400	−0.104	**0.749**	0.014
Flagellum (ant I)	0.394	−0.298	**0.736**	−0.021
Aesthetasc number (ant I)	0.210	0.375	0.019	**−0.568**
Body width	0.029	0.226	0.324	**0.710**
Propodus length (per I)	**0.921**	0.008	−0.016	−0.183
Propodus width (per I)	**0.764**	−0.452	0.274	0.113
Dactylus setae row length (per I)	**0.779**	−0.069	0.382	−0.075
Dactylus length (per I)	**0.856**	−0.237	0.276	0.055
Merus longest spine length (per I)	−0.115	−0.296	**0.840**	0.097
Pereopod IV length	−0.303	**0.908**	−0.049	−0.052
Propodus arbitrary length (per IV)	−0.188	**0.902**	−0.299	−0.098
Propodus polygon length (per IV)	−0.055	**0.909**	−0.338	−0.066
Pereopod VII length	**0.849**	−0.095	−0.044	0.150
Exopodit area (ple III)	0.128	−0.153	−0.123	**0.797**
Eigenvalue	4.01	3.13	2.43	1.57
Explained variance (%)	28.66	22.44	17.39	11.18

Note

Eigenvalues and explained variance (as percentage of the total variance) are also shown. Component loadings higher than 0.55 are in bold font.

Abbreviations: ant, antenna; per, pereopod; ple, pleopod.

The second PC (17.7% of variation) correlated positively with the characters measured on pereopod IV (total length, arbitrary and polygon length of propodus IV). The LMM on PC2 revealed significant habitat and sex effects, and a significant habitat ×sex interaction (habitat: *F*_1, 13_ = 19.50; *p* = .001; sex: *F*_1, 640_ = 127.81; *p* < .001; habitat ×sex: *F*_1, 640_ = 49.70; *p* < 0.001; Figure 4b). Cave populations had higher scores than surface populations, females had higher scores than males, and sexual dimorphism was more pronounced in cave than in surface populations.

The third PC (10.2% of variation) correlated positively with the length of the longest spine on the merus of pereopod I, and the peduncular and flagellar length of the antenna I. The LMM on PC3 revealed significant habitat and sex effects (habitat: *F*_1, 13_ = 48.78; *p* < .001; sex: *F*_1, 640_ = 20.02; *p* < .001; habitat ×sex: *F*_1, 640_ = 0.57; *p* = .45; Figure 4c). Surface populations had higher scores than cave populations and males had higher scores than females.

The fourth PC (9.9% of variation) correlated positively with body width and the pleopod III exopodit area and negatively with the number of aesthetascs on antenna I. The LMM on PC4 revealed a significant sex effect (habitat: *F*_1, 13_ = 0.29; *p* = .60; sex: *F*_1, 639_ = 79.71; *p* < .001; habitat ×sex: *F*_1, 639_ = 1.53; *p* = .22; Figure 4d). Females had higher scores than males. The random effect of population nested in habitat was significant in all four LMMs (all Wald Z > 2.52; all *p* < .016).

### 3.2 Approach 2: Multivariate linear model followed by linear mixed models

All effects in the mLM were highly significant (habitat: Wilk's α_17, 622_ = 0.09; *p* < .001; sex: Wilk's α_17, 622_ = 0.32; *p* < .001; habitat ×sex: Wilk's α_17, 622_ = 0.46; *p* < .001; population [habitat]: Wilk's α_221, 6489_ = 0.004; *p* < .001; body length: Wilk's α_17, 622_ = 0.09; *p* < .001). The results from the subsequent univariate LMMs on the 17 original variables together with the estimated marginal means (i.e., least‐squares means) and their standard errors are reported in Table 3. Below we present the results in the order in which the traits loaded to the PCs (see above).

**TABLE 3:** Results of the trait‐by‐trait linear mixed models. *F*‐values with their degrees of freedom are shown in parentheses

Trait	Habitat	Sex	Habitat*sex	Body length	Cave / female	Cave / male	Surface / female	Surface / male
Ped length (ant I)	24.21*** (1, 131)	82.43*** (1, 632)	0.04 (1, 641)	2362.69*** (1, 485)	80.26 ± 1.19	85.12 ± 1.17	87.39 ± 1.01	92.41 ± .0.96
Flag length (ant I)	56.71*** (1, 122)	129.04*** (1, 636)	0.82 (1, 640)	1375.52*** (1, 502)	73.20 ± 1.65	81.93 ± 1.63	89.06 ± 1.39	96.79 ± 1.34
Aesth number	3.85 (1, 131)	12.51*** (1, 647)	0.01 (1, 640)	70.13*** (1, 573)	3.76 ± 0, 16	3.97 ± 0.15	3.38 ± 0.13	3.59 ± 0.13
Longest aesth length	2.92 (1, 131)	4.94* (1, 646)	2.97 (1, 640)	50.25*** (1, 571)	4.54 ± 0.12	4.59 ± 0.12	4.22 ± 0.10.	4.39 ± 0.10
5^th^ article (ant II)	12.97* (1, 131)	65.31*** (1, 650)	43.23*** (1, 647)	1895.84*** (1, 645)	62.12 ± 1.66	67.89 ± 1.66	56.70 ± 1.38	58.10 ± 1.35
6^th^ article (ant II)	9.58** (1, 131)	88.86*** (1, 649)	69.13*** (1, 639)	1562.50*** (1, 650)	94.62 ± 3.20	106.28 ± 3.19	86.61 ± 2.64	89.01 ± 2.61
Body width	2.66 (1, 13)	17.14*** (1, 651)	0.02 (1, 639)	3718.30*** (1, 623)	242.13 ± 4.24	236.31 ± 4.23	250.75 ± 3.54	245.19 ± 3.46
Prop length (per I)	4.26 (1, 129)	764.00*** (1, 648)	9.69* (1, 640)	2915.95*** (1, 583)	63.22 ± 1.17	74.35 ± 1.16	59.16 ± 0.98	72.36 ± 0.95
Prop width (per I)	13.98** (1, 128)	621.08*** (1, 651)	67.46*** (1, 639)	1757.07*** (1, 628)	31.62 ± 1.39	39.78 ± 1.38	35.51 ± 1.16	49.02 ± 1.13
Dact sate row length (per I)	5.09* (1, 159)	353.29*** (1, 584)	0.06 (1, 642)	1784.95*** (1, 323)	34.24 ± 0.52	39.87 ± 0.51	35.71 ± 0.45	41.22 ± 0.42
Dact length (per I)	4.38 (1, 129)	478.47*** (1, 632)	2.86 (1, 641)	1907.05*** (1, 483)	53.19 ± 1.00	62.57 ± 0.99	55.17 ± 0.85	65.73 ± 0.81
Merus longest spine length	156.26*** (1, 123)	0.17 (1, 613)	0.14 (1, 641)	511.21*** (1, 406)	20.96 ± 0.53	21.14 ± 0.52	29.08 ± 0.45	29.11 ± 0.43
Per IV length	11.61* (1, 129)	142.70*** (1, 649)	14.43*** (1, 639)	1786.49*** (1, 650)	356.29 ± 7.51	329.79 ± 7.49	318.57 ± 6.20	302.24 ± 6.12
Prop arbitrary length (per IV)	45.57*** (1, 131)	270.02*** (1, 651)	190.92*** (1, 639)	615.68*** (1, 640)	77.09 ± 1.79	63.37 ± 1.79	56.42 ± 1.49	53.30 ± 1.46
Prop polygon length (per IV)	63.71*** (1, 132)	163.46*** (1, 651)	199.11*** (1, 639)	1035.11*** (1, 621)	80.19 ± 1.48	68.82 ± 1.47	60.12 ± 1.23	59.06 ± 1.21
Per VII	0.09 (1, 130)	272.57*** (1, 649)	2.68 (1, 639)	2606.18*** (1, 649)	553.40 ± 17.48	629.55 ± 17.44	551.86 ± 14.45	617.51 ± 14.25
Exopodit (ple III)	0.002 (1, 130)	0.60 (1, 644)	24.26*** (1, 638)	1530.47*** (1, 648)	152.96 ± 17.06	160.53 ± 17.04	161.58 ± 13.99	149.98 ± 13.92

Note

* denotes *p* < .05, ** denotes *p* < .01, *** denotes *p* < .001. The random effect (population) was always significant (all *p* < .029). Estimated marginal means (= least‐squares means) and corresponding standard errors are presented separately for males and females from cave and surface habitats.

Abbreviations: aesth, aesthetascs; ant, antenna; dact, dactylus; flag, flagellum; ped, peduncle; per, pereopod; ple, pleopod; prop, propodus.

The trait‐by‐trait analyses of the length and width of the propodus of pereopod I, the setae row length and the length of the dactylus of the same appendage, and the length of pereopod VII (traits loading on PC1) revealed concordant, but more variable results than the analysis of PC1. Results on pereopod I propodus length were fully congruent with results on PC1: males > females and sexual dimorphism was stronger in surface than in cave populations. In case of propodus width, patterns were similar, but we also found a significant habitat effect: surface populations had wider propodus than cave populations. In dactylus setae row length, we found a habitat effect similar to that of propodus width and a sex effect similar to that in PC1, but no habitat‐dependent sexual dimorphism. In pereopod I dactylus length and pereopod VII length, we only found sexual dimorphism similar to that in PC1.

The trait‐by‐trait analyses of total length of pereopod IV, arbitrary, and polygon length of propodus of pereopod IV (traits loading on PC2) revealed the exact same pattern as found in the PCA‐based analysis: cave populations > surface populations, females > males and sexual dimorphism being stronger in cave than in surface populations.

Analyzing the length of the spine on pereopod I merus and peduncular and flagellar length of antenna I (traits loading on PC3) revealed the same result for antenna I traits as we found for PC3: surface populations > cave populations and males > females. In case of the spine length, only a significant habitat effect similar to that in PC3 was detected: surface populations > cave populations.

Results on body width, exopodit area of pleopod III, and aesthetascs number of antenna I (traits loading on PC4) revealed the same pattern for body width and aesthetascs number to the one found in PC4: females had wider bodies than males, while males had more aesthetascs than females (negative loading on PC4). For exopodit area, only habitat ×sex interaction was significant. In caves, males, while in surface populations, females had larger exopodits.

Because the lengths of the 5^th^ and 6^th^ articles of antenna II loaded on more than one PC, they were excluded from the PCA (Norman & Streiner, 2008; Tabachnick & Fidell, 2013). In the trait‐by‐trait analysis, we found significant habitat and sex effects together with a significant interaction in case of the articles of antenna II: males > females; cave populations > surface populations and sexual dimorphism was stronger in cave than in surface populations. The length of the longest aesthetascs on antenna I was also removed from the PCA because it did not load on any of the PCs. In the trait by trait analysis, the aesthetascs length showed significant sexual dimorphism: males > females.

## 4 DISCUSSION

Caves can be seen as islands, with markedly different environmental conditions than the surrounding surface habitats, while themselves being similar (Mammola, 2019). They are isolated in most cases; hence, they offer a natural experiment to study parallel evolution (Pipan & Culver, 2012). Cave‐specific adaptations were recognized early (Christiansen, 1962, 1965) and, in some model species, including *A. aquaticus*, the study of troglomorphic adaptations reached the level of genomics (e.g., Carlini & Fong, 2017; McGaugh et al., 2014; Pérez‐Moreno et al., 2018; Protas & Jeffery, 2012; Re et al., 2018). However, comprehensive studies on morphological changes during the course of cave adaptation including many independent populations and large sets of functional traits, both needed for revealing dominant parallelisms, are scarce (Culver et al., 1995; Prevorčnik et al., 2004; Wilkens, 1988). Further, studies targeting how sexual dimorphism is affected during adaptation to the cave environment are lacking. Here, by studying both sexes from six cave and nine surface populations of the *A. aquaticus* species complex, we found various examples of parallel morphological evolution in caves and—besides the well‐known sexual dimorphism—habitat‐dependent sexual dimorphism. Below, we discuss the patterns following our main questions.

### 4.1 Habitat‐level divergence – parallel evolution

Habitat‐dependent selection acts on different aspects of species’ biology. Past studies mainly questioned how transition to dark environment affects organismal perception of the environment and how sensory equipment responds to these changes (Culver et al., 1995; Culver & Pipan, 2009), while other aspects of species’ ecology such as feeding strategies often remained unaddressed. Here, we discuss changes of functional morphology explainable by habitat‐driven natural selection.

Enhancement of extra‐optic sensing in subterranean habitats presumably leads to elongation of appendages, or sometimes only the distal articles of the appendages (Culver et al., 1995; Culver & Pipan, 2009; Prevorčnik et al., 2004). Our study provided mixed evidence for this so‐called elongation hypothesis. Cave populations have relatively longer antenna II (mechanosensory function) and pereopod IV (mechanosensory and sex‐dependent function, see below) than surface populations. Moreover, we found significant elongation not only in pereopod IV total length, but also the elongation of the propodus of pereopod IV (distal article) itself was significant. In sharp contrast with the elongation hypothesis, antenna I was shorter in cave populations (see also Prevorčnik et al., 2004). This appendage carries chemosensory aesthetascs that might be the targets of selection. However, neither the length of the longest aesthetascs nor the number of aesthetascs differed between surface and cave populations, grounding a premise that chemosensory function remained intact in this respect. It is possible that in caves in general with their simple communities (negligible predation and interspecific competition), enhancement of chemical sensing is less important for the species (but see sexual dimorphism results below suggesting its more pronounced importance for males). Further, assuming that mechanosensory performance is of high importance in the caves colonized by *A. aquaticus* and mechanosensory efficacy is indeed affected by the length of the appendage bearing the sensors, the primarily chemosensory antenna I might be in energetic or developmental trade‐off with the primarily mechanosensory antenna II.

Selection may also affect feeding habits. Surface *A. aquaticus* are detritivores. Organic debris in caves is presumably of low quality in comparison with biofilm (Poulson, 2012), thus scrapping of biofilm might be a relatively more important feeding strategy for cave populations (Francois et al., 2016) especially in closed systems such as Movile‐ and Molnár János Cave (Herczeg et al., 2020; Sarbu et al., 1996). We found that cave populations had narrower propodus (pereopod I) than surface populations, irrespective of sex. Since propodus is used in feeding (Bertin et al., 2002), it is conceivable that cave populations with their slender propodus need to handle less durable or smaller food items than surface populations, although behavioral evidence for this idea is needed.

The increasing oligotrophy in caves might also explain some changes not related to feeding biology. Cave populations had relatively shorter seta row on the dactylus (pereopod I) than surface populations. These setae are used for grooming antenna II. A possible explanation is that the organic matter—that might adhere to the antenna II—is more abundant in the surface habitats; therefore, a longer setae row is beneficial for cleaning.

Finally, cave communities are simpler, with fewer predators (Gibert & Deharveng, 2002). Assuming an antipredator function of spines in general (Boeing et al., 2006; Jugovic et al., 2010; Weiss et al., 2012), the shortening of the spine on pereopod I merus in caves (see also Sket, 1985) can be explained by a release of positive selection, stemming from the low or negligible predation pressure in caves and is in agreement with the decreased tendency for shelter‐seeking observed in some cave populations of *A. aquaticus* (Fišer et al., 2019; Romero, 2011).

Taken together, morphological changes in the course of adaptation to cave environment can be explained by various selection forces. That saying, we stress that explicit experimental evidence for trait function, heritability, and their link with fitness is mostly lacking. On the other hand, invoking multiple aspects may explain deviations from the expected pattern, as subterranean habitats also differ from surface habitats in other environmental factors (and their mutual interactions) than darkness, leading to different phenotypes. An example is the limited support for the elongation hypothesis. In previous studies on *A. aquaticus*—targeting a smaller set of traits/populations or one sex only—the lack of elongation had been reported for some traits (e.g., pereopod VII: Turk et al., 1996; Konec et al., 2015; antenna I: Prevorčnik et al., 2004). Our study supports the contention that even though elongation does occur, it is not a general rule regarding all appendages or appendage articles.

### 4.2 Sexual dimorphism

Male‐biased sexual dimorphism of the propodus and dactylus of pereopod I, which forms one mechano‐functional grabbing unit, is probably connected to the different functions in males and females. While pereopod I is generally used for feeding and grooming, the appendage is also used by males for grabbing the females during mating or for fighting with other males (Bertin et al., 2002), explaining the reported sexual dimorphism. Moreover, *A. aquaticus* males are bolder than females, and as such more exposed to predation (Harris et al., 2011). Therefore, it is possible that larger pereopod I might also help in antipredator behavior. We also found male‐biased sexual dimorphism in all antenna I traits (pedunculus and flagellum length, aesthetascs number, and the length of the longest aesthetasc). Considering the aesthetascs’ chemosensory function (Heimann, 1984), the male‐biased sexual dimorphism in antenna I traits can be explained by the importance of chemical information in male mate searching (Ridley & Thompson, 1985; Vesakoski et al., 2008) or it can help the detection of predators, which can be more important for the generally bolder males (Harris et al., 2011). The male‐biased sexual dimorphism in antenna II (mechanosensory function) was already reported from surface populations (Blasdent, 1965) and can be explained similarly as the sexual dimorphism in antenna I. Pereopod VII is associated with locomotor functions; therefore, longer pereopod VII suggests better walking performance (Bertin et al., 2002). Harris et al. (2011) found that males are more active in general than females and emerge earlier from shelter after disturbance, probably trading safety for advantages in mating and feeding. Such sexual differences in behavior might explain the male‐biased sexual dimorphism of pereopod VII reported here.

We found female‐biased sexual dimorphism in pereopod IV. This trait has different roles in males and females. Pereopod IV has a role in precopulatory mate guarding in male *A. aquaticus*, while it has a locomotor function in females (Needham, 1942). After seeing male‐biased sexual dimorphism in numerous traits connected to male mating behavior, one would expect a similar pattern here. However, it is possible that the mate guarding function is not depending on size, rather on shape in this case (propodus of pereopod IV is more curved in males, Verovnik et al., 2009), while effective locomotion is positively affected by appendage length increase. Therefore, it is possible that males are trading locomotion for mate guarding, resulting in female‐biased sexual dimorphism. We found female‐biased sexual dimorphism in body width too. Relatively wider bodies of females can be explained by fecundity selection, because wider body might allow the development of more and/or larger eggs, and the nursing of more offspring in the marsupium (Ridley & Thompson, 1979; Vick & Blum, 2010).

Sexual dimorphism is typically explained by sexual selection acting on males (traits important in male–male agonistic behavior or in female mate choice) and/or fecundity selection acting on females (traits important for developing more or larger offspring), while a somewhat less supported explanation revolves around intersexual resource partitioning (e.g., natural selection causes divergent evolution between sexes in traits important in feeding, when sexes are utilizing different food sources) (Andersson, 1994; Clutton‐Brock, 2007; Fairbairn et al., 2007; Shine, 1989). Our results fit well to the sexual/fecundity selection explanations; however, experimental tests are needed to unambiguously support it. The hypothesis of intersexual resource partitioning seems unlikely, given that *A. aquaticus* are shredders and scrapers of biofilms and given that male and females have similar mouthparts (Verovnik et al., 2009). Nevertheless, we note that eliminating this hypothesis would require additional analyses of feeding behavior and stable isotopes.

### 4.3 Habitat‐level divergence in sexual dimorphism

One of the most salient findings of this study is the recognition that the degree of sexual dimorphism differs between surface and cave populations. This may indicate that the habitat shift affected sexual or fecundity selection.

Increased male‐biased sexual dimorphism in propodus (pereopod I) width, and to a lesser extent, length in surface populations suggests stronger sexual selection acting on surface than on cave males. The lower selection pressure in caves might stem from the generally lower densities of cave populations (Mammola et al., 2021). Lower densities decrease frequency of aggressive interactions between cave males, as shown in cave‐dwelling fish (Elipot et al., 2013), and may decrease dimorphism in pereopod I. At the same time, lower densities require enhanced mate‐finding abilities, resulting in elaborated antenna II (Thiel & Duffy, 2007). This explanation is further strengthened by the findings of Bertin and Cézilly (2005). According to their results, in surface *A. aquaticus* populations with low population density, the determinant of pairing success is the localization of mates; thus, males with longer antenna II had a higher pairing success. Our results showing increased male‐biased antenna II sexual dimorphism in caves is congruent with the previous results. Taken together, the patterns suggest that in surface populations, traits important in male–male agonistic behavior and mate guarding, while in caves, traits important in finding mating partners are more important for male reproductive success.

In the previous sections, we discussed cave elongation of pereopod IV irrespective of sex‐ and female‐biased sexual dimorphism of pereopod IV irrespective of habitat. Here, we argue that the increased female‐biased sexual dimorphism of pereopod IV in caves might be a result of combined habitat‐ and sex‐related differences. In males, the female‐holding function is probably shape‐dependent and thus the function restricts elongation, while there is no such constraint in females where pereopod IV has a locomotor and mechanosensory function. This sexual difference in functional constraints could result in the reported higher female‐biased sexual dimorphism in cave than in surface populations. With other words, natural selection for elongation can operate more freely on an appendage with a general function in one sex than on the same appendage with a specialized function in the other sex. Alternatively, in line with the previous results on the propodus of pereopod I, it is possible that sexual selection on male traits is weaker in caves than in surface habitats. Nevertheless, it is possible that there is a completely different mechanism behind the elongation of pereopod IV in cave males, where we assume that its holding function is the sole driving force. In low‐density cave populations finding, an adequately sized female for successful precopulatory mate guarding is less likely. Males with longer pereopod IV might utilize a broader range of female sizes and thus increase their chances for successful reproduction. In this case, cave females and males have longer pereopod IV than their surface counterparts, but the sexual dimorphism is more pronounced because different mechanisms are responsible for the elongation.

Interpreting the opposite sexual dimorphism in exopodit area of pleopod III between cave and surface populations is particularly challenging, because of the trait's dual function. The general function of crustacean pleopods is swimming and respiration (Alexander et al., 1995). In *A. aquaticus*, pleopod III presumably does not take part in respiration (but see above and Prevorčnik et al., 2009). Needham (1938) states that *A. aquaticus* is incapable of swimming, and we agree that swimming in a classical sense is rarely performed by this isopod. Nevertheless, pleopod III helps the animals’ locomotion by propulsion. This appendage also helps the circulation of the water under the abdomen, which most probably affects the oxygenation of the respiratory and osmoregulatory organs as well as the eggs in the marsupium, similarly to other crustaceans (Dahl, 1977). We speculate that in cave males large exopodits are important for increased locomotor performance needed for mate searching in the low‐density populations, while in surface females, large exopodits are important for water circulation around the egg‐bearing marsupium during periods of low oxygen, which are often present in their native surface habitats.

Taken together, we found that in most cases, sexual dimorphism was biased toward the same sex in both cave and surface habitats, only the magnitude of dimorphism being different. These patterns suggest that the selective forces creating sexual dimorphism are similar in both habitats, only their relative strength being different. In cases where a pure habitat effect was also detected, we can conclude that evolutionary shifts occurred parallel between the sexes, but with different intensity. However, in one case, where sexual dimorphism was opposite between cave and surface habitats, we reject parallel evolution of the sexes and support highly divergent sex‐specific selective forces.

## 5 CONCLUSIONS

Our large‐scale study involving 656 individuals from six cave and nine surface populations of the *A. aquaticus* species complex revealed a large set of biologically relevant patterns. We found cave—surface habitat divergence in 10 out of 17 morphological traits, suggesting parallel morphological evolution during the course of cave adaptation in antenna I, antenna II, pereopod I, and pereopod IV. The elongation hypothesis—the elongation of structures bearing extra‐optic (chemosensory, mechanosensory) sensors in the absence of light (Christiansen, 1961)—was supported in the antenna II with mechanosensory functions and pereopod IV with general locomotor, mechanosensory, and special female‐grabbing functions. However, elongation was not a general trend. Similarly to Prevorčnik et al. (2004), we found shortening of antenna I (chemosensory functions) in caves, while other traits’ habitat divergence could not be linked clearly to elongation. We conclude that appendage elongation in caves is highly function‐specific. Although the presence of parallel evolution has been detected in various cave‐dwelling taxa (Jones et al., 1992; Strecker et al., 2012), it has also been proven that closer examinations at the population level can reveal deviations from the general pattern (Fišer et al., 2019; Konec et al., 2015). In our case, population variation within habitat type was statistically significant for every studied trait, which opens future venues to understand the fine details of between‐cave variation.

Sexual dimorphism was present in all traits, but the length of the longest spine on merus of pereopod I. In most cases, it could be explained by sexual selection acting on males, particularly through increasing mate searching and mate guarding success, but in some instances, selection for female fecundity or nursing behavior might be responsible. In eight cases, sexual dimorphism was habitat‐dependent, suggesting varying strength of sexual selection between habitats or a conflict between sexual selection and other sources of environmental selection. Typically, sexual dimorphism showed the same direction and differed between cave and surface habitats in magnitude only. However, in one case the direction of sexual dimorphism was opposite between the two habitats. These patterns draw attention to the fact that the strength of sexual selection might vary considerably between habitats and thus one‐population sexual dimorphism studies or one‐sex habitat comparisons can be highly misleading.

## CONFLICT OF INTEREST

The authors declare no conflict of interest.

## AUTHOR CONTRIBUTIONS

**Gergely Balázs:** Conceptualization (equal); data curation (equal); investigation (equal); methodology (equal); validation (equal); visualization (lead); writing—original draft (equal); writing—review and editing (equal). **Anna Biró:** Data curation (equal); investigation (equal); methodology (equal); software (equal); validation (equal); visualization (supporting); writing—original draft (equal); writing—review and editing (equal). **Žiga Fišer:** Investigation (equal); methodology (equal); writing—review and editing (equal). **Cene Fišer:** Conceptualization (equal); funding acquisition (equal); investigation (equal); methodology (equal); project administration (supporting); resources (equal); supervision (supporting); validation (equal); writing—review and editing (equal). **Gábor Herczeg:** conceptualization (equal); funding acquisition (equal); investigation (equal); methodology (equal); project administration (lead); resources (equal); supervision (lead); validation (equal); writing—review and editing (equal).

## Supporting information

Supplementary Material

## Data Availability

The data are available on Dryad doi (https://doi.org/10.5061/dryad.kwh70rz4j).
