# Supplementary material for: Parallel morphological evolution and habitat‐dependent sexual dimorphism in cave‐ vs. surface populations of the Asellus aquaticus (Crustacea: Isopoda: Asellidae) species complex
Source: Ecol Evol. 2021 Oct 20;11(21):15389–403. doi: 10.1002/ece3.8233 (PMC8571603; doi:10.1002/ece3.8233)
Supplement: Supplementary file 1 — Supplementary Material [file ECE3-11-15389-s001.docx]

**Sampling sites**

*Slovenia and Italy*

Eight sites were sampled on the North-Western part of Dinaric Karst. Six sites originated from Slovenia and two from Italy, altogether four cave and four surface populations forming four cave-surface population pairs. The first three population pairs, *PIV*-PLA, *ZEL*-ZEL, *LAB*-TIM, inhabit (pairwise) the same sinking river continuum of three rivers Pivka, Rak and Reka (Timavo), respectively. Animals exhibiting surface-related morphology are found in the surface stretches, and animals exhibiting cave-related morphology reside in the subterranean stretches. As there is no physical barrier between the surface and cave populations within these population pairs, occasionally surface individuals can be found in the caves and the other way around, especially after floods. In case of the last population pair (*KRS*-LJB), *A. aquaticus* is absent from the surface stretches of the sinking river (Verovnik et al., 2009), therefore, the closest known surface population was sampled.

The Planina Polje and Cerknica Polje surface populations are from typical karst poljes, sampled in the surface stretch of a sinking river. The Ljubljana Moors population was collected from an irrigation ditch in the typical marsh habitat common for the area south of Ljubljana, while the sampling of Timavo Spring population was at the resurgence of the sinking river Reka (Timavo). The four caves are ‘typical’ karst caves in the sense that they have stable low temperature (12°C) all year around, and animals in these caves rely on external food sources. In their study Konec et al. (2015) estimated that the most recent common ancestor in case of the Slovenian populations was 1.3 to 0.2 million years ago.

*Hungary*

Four populations were sampled from Hungary. The sampling sites of the Molnár János Cave (*MJ*) and Malom Lake (MT) population pair are situated at the foothills of the Buda Mountains in Budapest. Molnár János Cave is a 7 km long water-filled hydrothermal cave (Leél-Össy, 2017). At the cave entrance, a springlake (Malom Lake) is formed by the outflowing water. The third Hungarian sampling locality is the Csömör Stream, which is situated in the suburb of Budapest. The stream at the sampling point is concrete bedded with low water discharge. This stream from the source until the sampling point flows through inhabited areas and agricultural fields, therefore - similarly to other steams flowing through Budapest (Gombás et al., 2014) - the contamination level is relatively high compared to our other sampling places. The fourth site is the Dunakeszi Peat Moor, which is situated on the outskirts of Budapest. The moor is a stagnant water lake with low oxygen level and thick peat layer.

Csömör Stream and Dunakeszi Peat Moor represent typical surface habitats, being exposed to natural light and temperature fluctuation typical to the region. In the Malom Lake, the outflowing thermal karst water from the Molnár János Cave creates relatively high (22 ºC), stabile temperature all year long, while its’ light regime is typical to the region. As the water filling up Malom Lake is directly flowing out of the cave, the temperature and chemical composition of the water is almost identical to the caves’ (Kender, 1939). The *A. aquaticus* specimens collected from Malom Lake are typical surface animals with pigmentation and eyes. The Molnár János Cave population (which is known to be the only subterranean *A. aquaticus* population in Hungary) lives in relatively stable cave environment (Herczeg et al., 2020). It shows typical cavelife-related changes such as loss of pigmentation and eye degeneration (Pérez-Moreno et al., 2017). It has been genetically isolated for at least 60.000 years from the surface population inhabiting the Malom Lake, despite the lack of evident physical barriers (Pérez-Moreno et al., 2017). To our knowledge the amount of water discharged from the spring of the Molnár János Cave shows very weak correlation with surface precipitation (Erőss et al., 2006; Bodor et al., 2015). Therefore, it is highly likely that organic material of surface origin is not present in the cave. Accordingly, the population inhabiting the cave feeds on food of autochthonous origin mostly consisting of bacterial mats (Herczeg et al., 2020). Hence, Molnár János Cave is not the typical cave with low temperature and varying amounts of allochthonous food.

*Romania*

The three Romanian sampling sites are located in Dobrogea region at the close vicinity of Mangalia city. The hydrothermal Movile Cave System represents an energy rich, mesothermal habitat with a constant water temperature of 21°C (Sarbu & Popa, 1992). Food sources in the Movile Cave ecosystem are autochthonous and based on chemoautotroph bacterial production (Sarbu et al., 1996). The aquifer of Movile Cave extends beyond the cave itself and can be reached through wells (Sarbu et al., 2019). In these wells *A. aquaticus* is genetically and morphologically identical to ones in the cave but specimen collection is easier. Therefore we sampled a hand dug well (named after the street where it is located) which is known as Dimitru Ana Well. The population inhabiting Movile Cave is showing typical troglomorphic features. The second sampling site is an open water thermal lake called Kara-Oban, which is the part of the same thermal groundwater regime as the Movile Cave. Accordingly, the chemical composition of the lake is similar to the cave systems’ with high hydrogen sulphide and methane content which is typical to the region (Onac & Drăgușin 2017). The annual water temperature changes in the lake are slightly buffered by the thermal inflow, while the light regime is typical to the region. The population living in Kara-Oban Lake is categorized as a typical surface population. The Movile Cave (*CA*) population and the Kara-Oban lake (KO) population creates a pair as they are hydrologically connected. The third sampling site is the Baile Turcesti Spring. This site was chosen because the water of the spring is not connected to the thermal water system but fed by water of meteoric origin (derived from precipitation), which is exceptional in the area. The water temperature of the spring is stable all year round (14.5 °C) and the turbidity is extremely low. The population living in Baile Turcesti Spring (TB) is a typical surface population.

The Romanian subterranean population is genetic subgroup of the surface populations of the area with no ongoing gene flow between them (Konec et al., 2015). The most recent common ancestor of the surface and subterranean ecomorphs is dated between 3.8 to 0.2 million years ago (Konec et al., 2015).

References

Bodor P, Erőss A, Mádlné-Szőnyi J, Kovács J. 2015. A csapadék hatása a rózsadombi források utánpótlódási és megcsapolási területén, *Földtani Közlöny* 145(4): 385–396.

Erőss A, Mádl-Szőnyi J, Mindszenty A, Müller I. 2006. Conclusions from a negative tracer test in the urban thermal karst area, Budapest, Hungary. In: Tellam JH, Rivett MO, Israfilov RG, eds. *Urban Groundwater Management and Sustainability*. Springer, 289–299.

Gombás Á, Sárközi E, Kardos L, Angyal Zs. 2014. Három budapesti kisvízfolyás kémiai vízminőségének vizsgálata a területhasználat tükrében, *Economica* 3: 69–75.

Herczeg G, Hafenscher VP, Balázs G, Fišer Ž, Kralj-Fišer S, Horváth G. 2020. Is foraging innovation lost following colonization of a less variable environment? A case study in surface- vs. cave-dwelling *Asellus aquaticus,* *Ecology and Evolution* 00: 1–9.

Kender J. 1939. A Szent Lukács-fürdő tavának limno-biológiai vizsgálata, *Palaestra Calasanctiana* 25: 1–24.

Konec M, Prevorčnik S, Sarbu SM, Verovnik R, Trontelj P. 2015. Parallels between two geographically and ecologically disparate cave invasions by the same species, *Asellus aquaticus* (Isopoda, Crustacea), *Journal of Evolutionary Biology* 28: 864–875.

Leél-Őssy SZ. 2017. Caves of the Buda Thermal Karst. In: Klimchouk A, Palmer AN, De Waele J, Auler AS, Audra P, eds. *Hypogene Karst Regions and Caves of the World,* Springer, 279–299.

Onac BP, Drăgușin V. 2017. Hypogene Caves of Romania. In: Klimchouk A, Palmer AN, De Waele J, Auler AS, Audra P, eds. *Hypogene Karst Regions and Caves of the World,* Springer, 257–267.

Pérez-Moreno JL, Balázs G, Wilkins B, Herczeg G, Bracken-Grissom HD. 2017. The role of isolation on contrasting phylogeographic patterns in two cave crustaceans, *BMC Evolutionary Biology* 17(247).

Sarbu SM, Popa R. 1992. A unique chemoautotrophycally based cave ecosystem. In: Camacho A, ed. *The natural history of Biospeology*, Madrid: C.S.I.C, 641–666.

Sarbu SM, Kane TC, Kinkle BK. 1996. A chemoautotrophically based cave ecosystem, *Science* 272: 1953–1955.

Sarbu, SM, Lascu C, Brad T. 2019. Dobrogea: Movile Cave. In: Ponta GML, Onac BP, eds. *Cave and Karst Systems of Romania*, Springer, 429–436.

Verovnik R, Prevorčnik S, Jugovic J. 2009. Description of a neotype for *Asellus aquaticus* Linné, 1758 (Crustacea: Isopoda: Asellidae), with description of a new subterranean Asellus species from Europe, *Zoologischer Anzeiger* 248: 101–118.
